# Sex-Related Differences in the Immune System Drive Differential Responses to Anti-PD-1 Immunotherapy

**DOI:** 10.3390/biom14121513

**Published:** 2024-11-27

**Authors:** Sonja Cotra, Mohammad Kohandel, Michelle Przedborski

**Affiliations:** Department of Applied Mathematics, University of Waterloo, Waterloo, ON N2L 3G1, Canada; kohandel@uwaterloo.ca

**Keywords:** systems biology, immune checkpoint inhibitors, sex-specific differences, personalized immunotherapy, anti-PD-1 immunotherapy

## Abstract

Immune checkpoint inhibitors, such as anti-PD-1 antibodies, represent a significant advancement in cancer immunotherapy, but their efficacy varies notably between individuals, influenced by complex biological systems. Recent evidence suggests that sex-related biological differences play a pivotal role in modulating these responses. This study uses a systems biology approach to examine how sex-specific differences in the immune system contribute to variability in the response to treatment. Our model extends previous frameworks by incorporating sex-specific parameters that reflect observed immunological distinctions. The results from the simulation studies align with our clinical observations, showing that on average, males exhibit a more robust response to anti-PD-1 treatment compared to females. Additionally, this study explores the potential of combination therapy with recombinant IL-12, revealing sex-specific differences in treatment efficacy. These findings underscore the need for personalized immunotherapy strategies that consider individual immunological profiles, including sex, to optimize treatment outcomes.

## 1. Introduction

Immune checkpoint inhibitors (ICIs) have been a promising avenue for cancer immunotherapy in the last few decades. Designed with high affinity for specific membrane-bound receptors, such as PD-1, PDL-1, and CTLA-4 [1], such antibodies block mechanisms that enable cancer cells to evade the anti-tumor activity of T-cells, which, in principle, enables the immune system to recognize and kill the malignant cells. Similar to other immunotherapies, the clinical success of ICIs varies greatly among patients and is unpredictable [2,3], which reflects the impact of patient-specific variability in immune response on treatment success. Immunotherapies targeting the PD-1/PDL-1 pathway have surpassed the 10% response rate ceiling typically associated with other immunotherapies [4], though they are subject to limitations imposed by a patient’s specific immune system and tumor microenvironment. Consequently, it is imperative to investigate how variability in the immune system can influence treatment response to better predict therapeutic outcomes for the average patient and to develop strategies for identifying which patients are good candidates for immunotherapy.

Pre-clinical studies and clinical trials using live human subjects are crucial for the development and refinement of dosing regimens for pharmaceutical interventions. However, they are expensive and time-consuming to conduct, and they do not necessarily provide insight into the range of responses that can be exhibited due to patient-specific variability. Systems biology is a cost-effective approach that enables the integration of experimental data with computational and mathematical modeling, allowing for in-depth analysis of the complex interactions that can drive variability in a biological system and lead to differences in treatment response. This is particularly important for the study of how variability in the immune system can affect responses to anti-tumor therapies, given the complexity of the immune system resulting from both positive and negative feedback loops between different cell subtypes and signaling molecules.

One major variable in anticancer immunity is sex hormones [5], which exert regulatory effects on both the cancer and immune cell populations [6]. Indeed, sex hormone receptors are over-expressed in many different cancer types, leading to the misregulation of several pathways related to growth and development. In breast tissue, for example, sex steroids sustain the stem cell population (in normal and malignant cells) [7] and they play a key role in the epithelial–mesenchymal transition (EMT), which leads to metastasis and invasion [8]. While the data are limited, they point to estrogen receptor β (ERβ) and progesterone receptor (PR) isoforms as the key drivers of these effects, while the role of androgen receptors (ARs) remains uncertain [7]. In contrast, in prostate cancer, it is generally accepted that tumor growth is promoted by ARs and inhibited by ERβ, though emerging data suggest that ERβ may have dual effects [9]. In immune cells, estrogen induces the Th2 response and T-cell homing, while androgens, such as testosterone, augment the Th1 response and the activation of CD8+ T-cells, leading to increased production of anti-inflammatory cytokine IL-10 [10]. In our previous work [11,12], we found significant sex-related differences in patient-derived distributions of cytokine levels and immune cell populations under both treatment and control conditions. Given the breadth and complexity of these differential regulatory effects, systems biology provides the optimal approach to study how sex-related differences in the immune system drive response dynamics for cancer immunotherapy.

Here, we extend our previously developed systems biology model for anti-PD-1 immunotherapy [11,12] to examine how sex-specific differences in the immune system contribute to variability in the response to treatment. The model simulations are consistent with previous clinical results [13,14], which showed that, on average, biological male cancer patients experience more favorable outcomes than female patients in response to anti-PD-1 immunotherapy. Indeed, due to known basal immune variations between sexes [15], as well as the historical exclusion of females from clinical trials [16], it is unsurprising that therapies developed with the male immune system in mind appear to prove less effective in the female population. Thus, the results from this in silico study strengthen the demand for research into the particular immune characteristics that influence the treatment response gap between males and females, which can further inform systems biology-based and clinical studies aiming to rectify it.

This manuscript is structured as follows. In Section 2.1, we describe the development of the mathematical model and simulated treatment protocols for both anti-PD-1 immunotherapy alone and in combination with recombinant IL12. We detail the integration of established immune cell interactions with ex vivo data sampled from a cohort of patients with head and neck squamous cell carcinoma. To this end, we first outline the literature review that was conducted to understand the known differences in the immune system between males and females. Then, in Section 2.2, we describe how these differences were incorporated into the mathematical model by changing key model parameters. We further describe how parameter sets representing “male” and “female” virtual patients were generated via Latin hypercube sampling. Afterwards, we provide greater detail for the simulation of combination therapy with recombinant IL12 in Section 2.3. Following simulations of anti-PD-1 immunotherapy, sex differences among the patient populations in the basal immune system are discussed in Section 3.1, and in Section 3.2, we assess the efficacy of the treatment, presented as the percentage of both the male and female virtual populations that responded favorably to treatment. In Section 3.3, we further investigate the differences between male and female virtual populations in response to combination therapy with anti-PD-1 immunotherapy and recombinant IL12. Finally, in Section 4, we conclude with a discussion of the results of the study and their implications for developing personalized approaches to immunotherapy.

## 2. Materials and Methods

### 2.1. Systems Biology Approach

**Figure 1:**
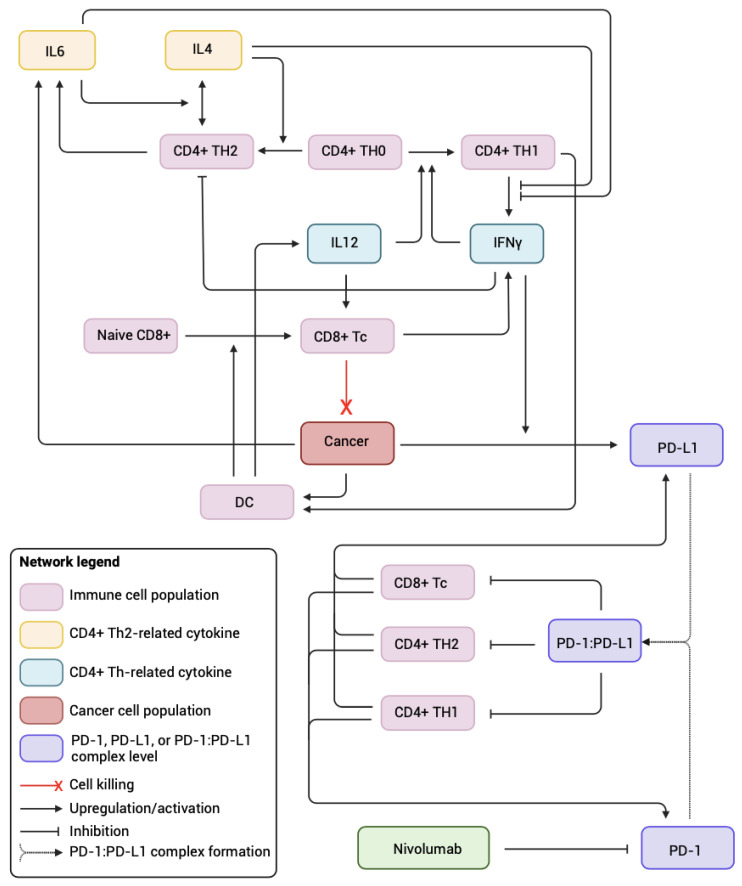
The molecular pathway for the previously established systems biology model used in this study. It displays the interactions between immune cell populations of CD4+ $T_{H0}$, $T_{H1}$; and $T_{H2}$ cells; CD8+ naive and cytotoxic T-cells; cytokines IL4, IL6, IL12, and IFNγ; dendritic cells; cancer cells; and PD-1 and PD-L1.

In previous work [11,12], we developed a multi-scale network describing the interactions [17,18,19,20,21,22,23,24,25,26,27,28] between key immune cell subtypes and cytokines that are relevant to immunotherapies targeting PD-1 receptors. This mathematical model was parameterized using a combination of literature data and temporal measurements of T-cell populations and cytokine secretion observed in a live ex vivo human system obtained from biopsies of patients with head and neck squamous cell carcinoma (HNSCC) at advanced and late stages. The mathematical model is comprised of 17 coupled ordinary differential equations and a total of 47 kinetic parameters. These equations were solved using the MATLAB ode15s solver (R2024a). Described by the pathway shown in Figure 1, with five key T-cell populations and four cytokines that are critical to the differentiation and activation of T helper cells, the model was used to simulate a 72 h treatment protocol in which 132 μg/mL of nivolumab was administered at t=0 h, t=24 h, and t=48 h with drug washout between each dose.

Patient parameter sets were generated using Latin hypercube sampling (LHS), with bounds supported by the literature [29,30,31,32], where possible, and the ex vivo data. Following nivolumab treatment simulation, each patient parameter set was given a “Class” value of 1 or 0, respectively, indicating response or nonresponse to the therapy. The Class of the patient parameter set was determined empirically by comparing the number of cancer cells after the three-day dosing schedule to the number of cancer cells after three days of no treatment. Specifically, the treatment was considered a success if the number of cancer cells on day three with treatment was less than the number of cancer cells after three days with no treatment, meaning that the treatment had slowed the tumor growth; in this case, the patients in this parameter set were deemed responders. We note that patient parameter sets that led to cancer regression after 3 days of no treatment were excluded from this analysis to avoid the inflation of response rates within a patient population. It is conceivable that, within the responder Class, there may be several subsets of response types. For example, some parameter sets might lead to a partial response, where the treatment minimally slows the tumor growth compared to an untreated case. For other parameter sets, there may be a more complete response, where the treatment actually causes cancer cell regression over time. Given these different types of response, it is important to note that patients deemed “responders” in this work may still have an increased cancer cell count after treatment compared to the initial cell count.

Creating virtual patient populations consisting of several hundred or thousands of patients enabled the examination of how variability in immune parameters affects the response to therapy. The assessment of the overall efficacy of the therapy for a particular population was performed by calculating the proportion of responders among the patients; this was referred to as the response rate of the given nivolumab therapeutic session.

### 2.2. Generating Male and Female Virtual Patient Populations

For several years, researchers have explored the differences and similarities between the male and female human immune systems. Notably, differences in T-cell populations and function [33,34], as well as cytokine production [35,36,37], have been reported between the sexes. Furthermore, clinical data have revealed that 55% of males have an inverted CD4:CD8 ratio (i.e., <1) compared to just 44% of females [38]. As another example, female lung cancer patients have, on average, a higher serum sPD-1 level of 105.3 pg/mL, compared to 75.17 pg/mL in their male counterparts [35]. Since the model in this study was developed using ex vivo data from biopsies of patients with HNSCC, serum levels of sPD-1 were not directly used to parameterize the model. However, as the goal was to explore the potential impact of previously reported sex-specific immune system differences on anti-PD-1 immunotherapy response, and differences in PD-1 expression have been reported for other types of cancer, PD-1 expression was taken to be sex-specific in the model. Moreover, to ensure that other potentially relevant sex-specific differences were properly reflected in the model, data and knowledge from the literature were carefully cross-referenced with the model parameters, enabling the identification of parameters that are likely to be sex-specific and could be leveraged to capture expected differences between male and female patients. The sex-specific differences captured by the model, along with the specific parameters that were altered to capture these differences, are summarized in Table 1.

To efficiently generate sex-specific patient parameter sets for further simulation and analysis, we built on the results from prior work [11,12], where a global sensitivity analysis identified the model parameters that most highly impact the treatment response rate. Specifically, we cross-referenced the most sensitive model parameters with those that are expected to exhibit sex-specific differences (Table 1) to pinpoint a subset of parameters, Ω, that were both influential on the model response rate and *potentially sex-specific*. We then altered the sampling ranges for this subset of parameters to generate sex-specific parameter sets, representing male and female virtual patient populations, such that the difference in response rates between the virtual populations was similar to what is observed clinically. In particular, parameter sets generated via Latin hypercube sampling using the nominal parameter ranges (see Table S2) were taken to represent the male virtual populations. The goal was then to alter the sampling ranges appropriately to generate female virtual populations that exhibited a ≈10–15% lower response rate, while keeping the parameters within biologically relevant ranges [13,14].

This was accomplished as follows. First, the sampling range for each parameter in Table 1 was individually subjected to a significant increase with respect to its nominal range, a virtual population of 1000 was generated via LHS, and the change in the response rate, relative to the nominal sampling range, was noted. Next, the sampling range for each parameter in Table 1 was individually subjected to a significant decrease with respect to its nominal range, and the response rate of the corresponding virtual population was noted. This was repeated for each individual parameter in the Table 1. In addition, the analysis was repeated for different factors applied to the sampling ranges, with the aim of identifying the degree that each sampling range must be altered to produce a significant change in the response rate compared to the nominal sampling range (i.e., virtual male population), as well as the directionality of the change. We observed that an increase or decrease by a factor of 50, that is, either a multiplication or a division of the nominal parameter sample range by 50, proved sufficient in providing insight into the sensitivities of the kinetic parameters. For example, a 50-fold increase in the sampling range for the net proliferation rate of $T_{H1}$ cells, while keeping all other parameter sampling ranges unaltered, led to a response rate that was roughly 19% lower than that of the virtual male population.

Due to the different natures of the parameters, not all of the parameters in Table 1 could simply be altered by multiplying or dividing their sampling ranges by 50. Specifically, some of the parameters correspond to a fraction of a total number of cells, and thus are defined as a value between 0 and 1. For this case, the sampling range was increased by raising just the lower bound of the sampling range to be arbitrarily close to the upper bound. Similarly, the sampling range was decreased for such parameters by lowering the upper bound of the sampling range to be arbitrarily close to the lower bound. For example, for any given fractional parameter, let *l* be the nominal lower bound from which it is sampled and *u* be the nominal upper bound such that 0≤l<u≤1. If we wanted to sample from a new range that is the bottom 10% of the original range, the lower and upper bounds of the new range, denoted $l_{b}$ and $u_{b}$, would be calculated as follows:

(1) $$\begin{array}{cc} l_{b} & =l\\ u_{b} & =0.1\times u \end{array}$$

Similarly, a new range $\left[l_{t},u_{t}\right]$ where $0\leq l_{t}<u_{t}\leq 1$, indicating the upper 10% of the nominal range [l,u], would be defined by:

(2) $$\begin{array}{cc} l_{t} & =u-0.1\times (u-l)\\ u_{t} & =u \end{array}$$

As the lower and upper 10% of a range was deemed sufficiently dramatic for a single-parameter sensitivity analysis, the LHS bounds of the fraction parameters were each subjected to changes detailed in Equations (1) and (2), seeking the aforementioned 10–15% effect on response rate.

Parameters that satisfied the required effect on the response rate when altered dramatically in accordance to their given parameter type were then deemed suitable for inclusion in the subset Ω, designating them as both sex-specific and pivotal to the response rate. With Ω cemented, the generation of female patient sets could begin. Unfortunately, little empirical data exist in the literature that can be used to identify distinct ranges of kinetic parameters for male and female virtual patients with a high degree of confidence. Non-identifiability in the model structure further compounds this limitation. Consequently, the approach used in this work focused on generating pseudo-random parameter sets that could accurately recapitulate the difference in the response rate to anti-PD-1 immunotherapy that is observed between male and female patients in the clinic [13]. To this end, the patient parameter sets that were obtained by sampling the ranges reported in Ref. [12] were taken to represent virtual male patients, and female virtual patient populations were obtained by altering the sampling ranges for the parameters in Ω. To accomplish this, a randomly generated factor was generated for both LHS bounds of each and every sex-specific parameter. Every one of these factors was used to either multiply or divide its particular bound for all parameters simultaneously, depending on the directionality of the sex difference. Due to the general absence of knowledge regarding particular ratios between the parameters in the male and female immune system, the random factors were kept within minimal ranges so as to not overstate the sex differences. While prior experimentation with individual parameter sensitivity used relatively large factors to induce a significant change in the response rate, altering multiple parameters together allows for achievement of the desired effect via much more modest adjustments, with random factors not exceeding 3.0.

For example, let parameters x,y∈Ω be sex-specific parameters such that the female immune system exhibits higher *x* values and lower *y* values compared to males. Let $0\leq l_{x}<u_{x}$ be the nominal (male) lower and upper LHS bounds for *x*, and $0\leq l_{y}<u_{y}$ be the nominal lower and upper LHS bounds for *y*. Supposing that the random factor to be applied to the parameters must be between *m* and *n* such that 1<m<n and m,n∈R, the female LHS bounds for *x* and *y* are determined using the rand() function in MATLAB [39]. When given an input i∈R, rand(i) will output a random number between 0 and *i*. Thus, a random number between *m* and *n* would be

(3) m+rand(n−m)

Then, the lower and upper LHS bounds for *x*, denoted by $l_{xf}$ and $u_{xf}$, respectively, would both be calculated by multiplying their male counterpart by said random factor, as represented by the equation below.

(4) $$\begin{array}{cc} l_{xf} & =(m+rand(n-m))\times l\\ u_{xf} & =(m+rand(n-m))\times u \end{array}$$

Conversely, since *y* represents a parameter with decreased values in females compared to males, the new LHS bounds $l_{yf}$ and $u_{yf}$ would instead be calculated by dividing the equivalent nominal bounds by a randomly generated factor, as illustrated by the following equation.

(5) $$\begin{array}{cc} l_{yf} & =\frac{1}{\left(m+rand(n-m)\right)}\times l\\ u_{yf} & =\frac{1}{\left(m+rand(n-m)\right)}\times u. \end{array}$$

To generate a given virtual patient population, the rand() function was run for each bound of each parameter individually, generating a unique output for each parameter bound. Thus, it should be noted that the factors multiplying *l* and *u* to create $l_{xf},u_{xf},l_{yf},$ and $u_{yf}$ are all distinct from one another.

The female virtual patient populations examined in this work were generated by applying the method described above to each parameter in the subset Ω, with each bound adjusted according to the nature of the sex difference described in Table 1. For a given virtual population, all randomly generated factors were sampled from the same range for every kinetic parameter. Importantly, since the sampling range for the random factors is taken to be the same for all parameters, this method may not accurately capture sex-specific differences that are quantitatively larger than others. However, the method was chosen for simplicity in the absence of direct empirical data on the sex-related differences for the specific relevant parameters.

Due to uncertainty regarding specific female parameter values in relation to the nominal (male) parameter set, multiple female populations were generated using six ranges in which the random factor for the alteration of kinetic parameters would be generated. In order of decreasing modesty, these ranges include 1.0–2.0, 1.1–2.5, 1.1–3.0, 2.0–2.5, 2.0–3.0, and 2.5–3.0. Each of these ranges was able to produce favorable results in regard to the 10–15% difference in response rate. Furthermore, the fraction parameters were also altered in various fashions to further increase the range of intensity by which the female parameters differ from the male parameters. Consisting of just the CD8+ naive T-cell fraction (TN8) and the CD8+ cytotoxic T-cell fraction (Tc), the fraction parameters experience alterations limiting both parameters to the bottom 80% of their respective nominal sampling ranges, and then both to the bottom 50%. Further extreme effects were experimented with by only applying lowered ranges to the CD8+ naive %, since the literature appears to suggest a lower response rate in females, starting with the bottom 50%, then the bottom 10%, and finally, the bottom 5% of the nominal sampling range. Altogether, a total of 30 female populations of 1000 patients each were generated for this analysis, as well as one male (defined with unaltered parameter sampling ranges) population of 1000 patients, one male population of 50,000 patients, and one “very” female population of 50,000 using the most extreme alterations on the fraction and the kinetic parameters.

### 2.3. Simulating Recombinant IL12 + Nivolumab Combination Therapy

Endogenous IL12 production critically regulates anti-PD-1 treatment potency, and recombinant IL12 (RIL12) has previously shown potential for amplifying the anti-tumor effects of PD-1 blockade in mouse studies [40]. Based on these findings, the administration of RIL12 in combination with nivolumab in a clinical setting may have a critical impact on treatment response and efficacy. Hence, in this part of the work, we focused our analysis on patients who were classified as nonresponders in simulations of nivolumab treatment alone, examining the rate of conversion of nonresponders into responders following in silico doses of RIL12 in combination with nivolumab. Thus, our investigation depended on the virtual populations of patients generated to simulate nivolumab monotherapy, specifically the Class identifier indicating each patient as a responder or nonresponder to monotherapy.

From there, all nonresponders identified via the aforementioned Class were extracted for examination and compiled in ascending order of the final cancer cell count after the 72 h nivolumab treatment protocol. That is, the nonresponders were organized according to how close they were to responding to nivolumab and thus how likely they would be to respond to the combination therapy. The top 100 nonresponders from this ordered list were then selected to undergo combination therapy simulations. Nearly identical to the nivolumab simulations, the combination therapy simulations included a dose of RIL12 administered prior to the first dose of nivolumab. After applying the combination treatment to each of the top 100 nonresponders, the percentage of those who responded to the combination treatment was calculated; this value was treated as a conversion rate from nonresponders to responders and gauged the efficacy of combination therapy with RIL12 on the patient population. Conversions were treated similarly in this analysis to those for the analysis of the response to nivolumab monotherapy, i.e., a response to combination therapy was defined as a decreased rate of cancer cell growth compared to an untreated case. In this regard, RIL12 combination treatment analysis included comparison with a simulation for each patient parameter set in which no treatment was administered whatsoever. The length of the simulation for the untreated case was taken to be the total treatment time for the combination therapy and thus depended on the administration time of the RIL12. The final cancer cell count was recorded and used to determine the Class of the patient parameter set: the patients in the parameter sets that led to a smaller number of cancer cells after treatment compared to an untreated case (over the same time interval) were taken to be responders.

## 3. Results

### 3.1. Sex-Specific Differences in Basal Immune System

**Figure 2:**
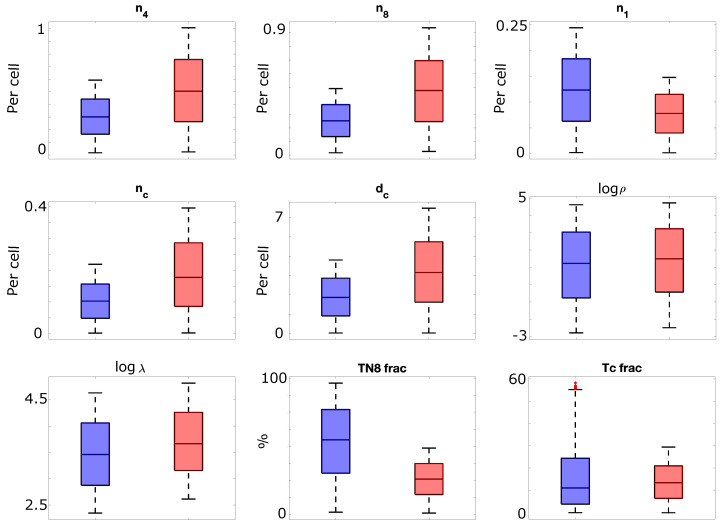
Box plots demonstrating the differences between male (blue) and female (red) virtual patients for each parameter in the subset Ω. The male virtual patient population was comprised of 1000 patients with parameters generated via Latin hypercube sampling of the parameters from the ranges summarized in Table S2. The female virtual patient population was comprised of 1000 patients generated via Latin hypercube sampling of the parameters, with the ranges of the parameters in Ω altered to represent the ranges expected for female patients. Specifically, parameters that do not represent a fractional cell count were multiplied by a random number between 1.1 and 2.0, generated individually for each parameter, while the naive and cytotoxic CD8+ T-cell fractions were limited to the bottom 50% of the sampling range used for male patients. For parameters ρ and λ, the log (base 10) of the parameter values is depicted.

Following the sensitivity analysis described in Section 2.2, a total of nine parameters were determined to be both sex-specific and influential on the response rate and thus comprise the subset Ω. Some parameters from Table 1, such as $p_{1-IFN}$ and $p_{2-4}$, were deemed insufficiently sensitive through the sensitivity analysis and were thus excluded from Ω (and the sex-based parameter changes). Others, including $n_{4}$, $d_{c}$, and especially λ, demonstrated a sufficiently significant impact on the response rate. In Figure 2, we plot the distribution of the parameters in Ω for populations of 1000 male and 1000 female virtual patients. As indicated in the boxplots in Figure 2, both the average value and range of each parameter are distinct between the male and female virtual populations, and they follow the trends reported in the literature. For example, the parameters $n_{4}$ and $n_{8}$, representing the proliferation of CD4+ and CD8+ naive T-cells, respectively, are higher in value in the female virtual population than the male, which is consistent with higher female CD4+ and CD8+ T-cell activity [15]. Similarly, higher CD8+ cytotoxic activity [33] is captured in female virtual patients compared to male virtual patients via increased values of parameters $n_{c}$ and $d_{c}$ in females, which are, respectively, the IL12-independent proliferation rate of cytotoxic T-cells and the rate of differentiation of CD8+ naive T-cells into cytotoxic T-cells. The CD4:CD8 ratio has also been reported to be higher in female patients than male patients [15]. This difference is captured in simulated patients by enforcing lower fractions of TN8 and Tc cells at baseline in female patients, which together comprise the fraction of T-cells that are CD8+ as opposed to CD4+. Finally, the per-cell expression of PD-1 and PD-L1 in male and female virtual patients follows the trends observed in the literature. Specifically, high PD-L1 expression is observed more frequently in tumors originating from female patients [36], which is consistent with the trends for λ in Figure 2. On the other hand, while the expression of PD-1 is generally higher on female CD4+ T-cells [35] and male CD8+ T-cells [37], the dominance of CD4+ and CD8+ T-cell activity in females, as discussed above, leads to a small overall increase in ρ, the per-cell PD-1 expression, in female virtual patients.

### 3.2. Sex-Specific Responses to Nivolumab

**Figure 3:**
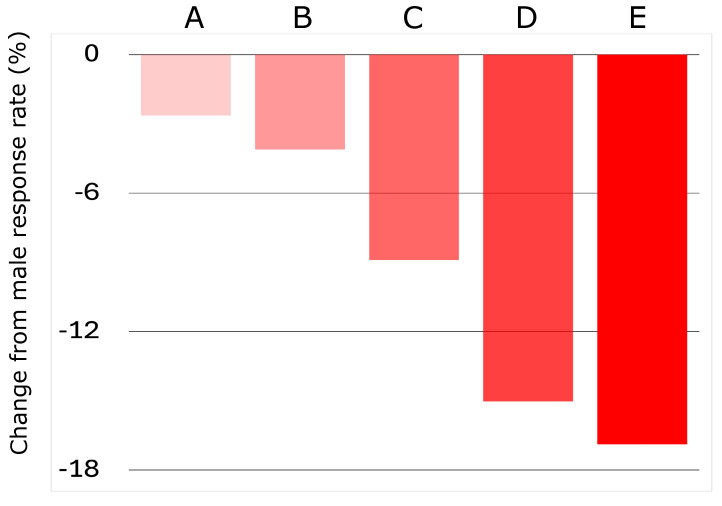
The differences in response rates for several female virtual patient populations (A–E) compared to the reference male population. All female populations were generated by multiplying the lower and upper bounds of the sampling ranges for each non-fraction sex-specific kinetic parameter in Ω by random factors in the range [1.1, 2.0]. The different populations were generated by sampling the initial naive CD8+ T-cell and CD8+ cytotoxic T-cell populations from different ranges. Specifically, population A was generated with the CD8+ naive % and CD8+ cytotoxic %, both confined to the bottom 80% of their LHS bounds. Population B features the same parameters further confined to the bottom 50% of the LHS bounds. For populations C, D, and E, only the CD8+ naive % LHS range was lowered to the bottom 50%, 10%, and 5%, respectively, while the sampling range for CD8+ cytotoxic T-cells remained equivalent to the male population.

The response rates to the 72 h nivolumab treatment protocol were recorded for 1 male and 30 female virtual populations, each comprised of 1000 patients, for a sex-based comparison. In general, female populations that were generated with the distributions of parameters depicted in Figure 2 proved less responsive to treatment than the male population, the latter exhibiting a response rate of approximately 56% in the simulations. In this work, the response rate was defined as the percentage of the total virtual population that were flagged as responders to the 72 h nivolumab treatment protocol. As displayed in Figure 3, female populations with minimal changes to the sampling ranges for parameters in Ω exhibited a fairly significant decrease in response to nivolumab, thus appearing to uphold the male bias in immunotherapy response as reported in the literature [13,14]. Female populations generated with more extreme differences in the sex-specific parameters Ω exhibited even larger reductions in response rate, with some populations having response rates up to 20% lower than the male virtual population.

**Figure 4:**
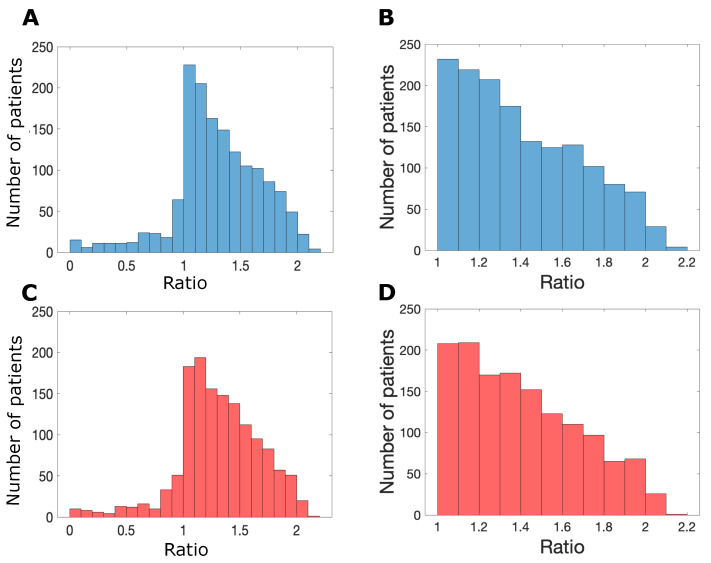
The distribution of the ratio of cancer cell count after the 72-h nivolumab treatment protocol to the initial cancer cell count. (**A**) Male responders, (**B**) male nonresponders, (**C**) female responders, (**D**) female nonresponders. The female population of patients was generated by multiplying the LHS lower and upper bounds by random factors in the range [1.1, 2.0], while limiting the naive and cytotoxic T-cell sampling ranges to the bottom 50% of the nominal range. Both male and female populations comprised approximately 1500 patients each.

As discussed in Section 2.1, we distinguished responders from nonresponders by comparing the ratio of the number of cancer cells after the three day nivolumab treatment schedule to the number of cancer cells after three days of no treatment. Therefore, the patient parameter sets are defined as responders when the treatment sufficiently slows the rate of cancer cell growth over time; however, in some cases, the cancer cell population regressed compared to the initial cell count. This behavior is visualized in Figure 4, which depicts the distribution of the ratio of cancer cell counts after nivolumab treatment to the initial cancer cell count. In both male and female populations of approximately 1000 patients, the figure illustrates that for most of the patients who were classified as “responders” (c.f. Figure 4A,C), the ratio was greater than one, indicating an overall increase in the cancer cell counts over time. Therefore, using a stricter definition of “responder” that requires regression of the cancer cell count over time, the response rate would be much lower. Indeed, populations of 1000 male and 1000 female patients (generated as described in Figure 2) exhibited response rates of 13% and 11%, respectively, when using the stricter definition of response.

### 3.3. Success of Simulated Recombinant IL12–Nivolumab Combination Therapy Depends on Patient Sex

We previously found [12] that combination treatment with recombinant IL12 (RIL12) administered prior to anti-PD-1 immunotherapy could improve the response rate compared to anti-PD-1 immunotherapy alone. Here, we analyzed whether male and female virtual patient populations exhibit different responses to this combination treatment and whether the optimal sequencing of the drugs depends on patient sex. In an attempt to capture a high degree of patient variability, during this analysis, we generated large populations of 50,000 virtual patients for each sex. Due to a general lack of knowledge regarding the impact of patient sex on the efficacy of recombinant IL12, we sought to exaggerate the sex-specific differences between the male and female patient populations. To this end, during the analysis, the nominal parameter ranges were used to generate the male population; to generate the female population, the lower and upper bounds for sampling the sex-specific kinetic parameters in Ω were scaled by random factors in the range [2.5, 3.0], and the TN8 fraction sampling range was restricted to the bottom 5% of its nominal range. We note that the male and female populations used for this analysis exhibited response rates of 54.69% and 33.08%, respectively, to the 72 h nivolumab monotherapy. With the female response rate approximately 21% lower than the male response rate, these larger simulations align well with the results discussed in Figure 3 and may thus be used for combination therapy analyses.

**Figure 5:**
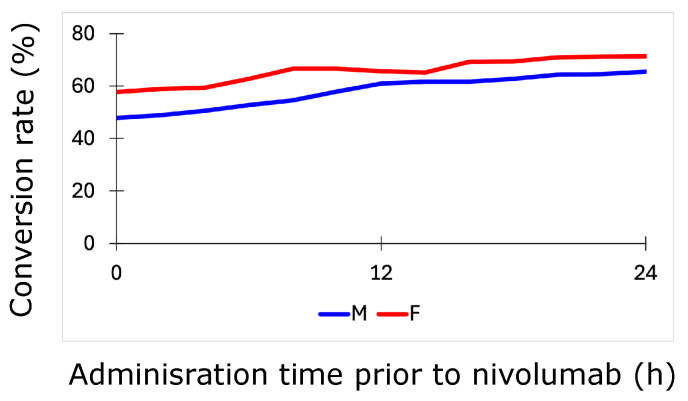
Simulations of 10 pg/mL recombinant IL12 for male (M) and female (F) patient populations with administration times up to 24 h prior to nivolumab. The conversion rate refers to the percentage of nonresponders to nivolumab alone who converted to responders following the addition of simulated RIL12 to the treatment schedule. The male patient results are denoted by the blue line, and the female patient results are denoted by the red line.

After both populations of patients were generated via Latin hypercube sampling, they were subjected to several combination treatment scenarios to elucidate the impact of the duration of pre-treatment with recombinant IL12 on the nivolumab treatment response. In the simulations, a dose of 10 pg/mL RIL12 was administered for different pre-treatment windows before nivolumab administration, and the results for both patient populations suggest that the increase in efficacy is approximately proportional to the length of the pre-treatment window (see Figure 5). Indeed, RIL12 treatment converted approximately 50% of males and nearly 60% of females to responders when administered at the same time as nivolumab, and these figures rose to about 60% and 70%, respectively, when RIL12 was administered 24 h prior to nivolumab.

**Figure 6:**
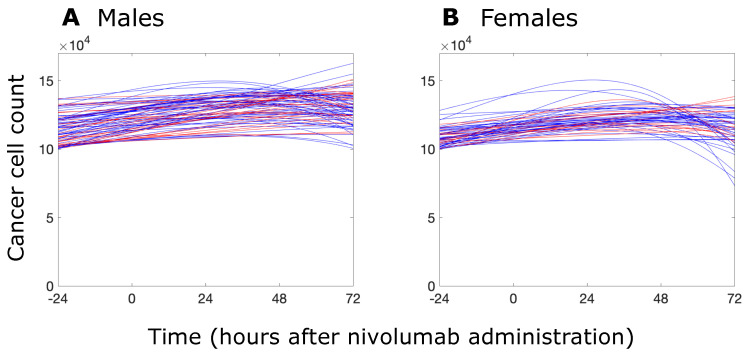
Simulated patient cancer cell counts in response to recombinant IL12 administered 24 h prior to nivolumab. Graph (**A**) represents the cancer cell count in a virtual population of 50,000 male patients, and graph (**B**) represents the same for a virtual population of 50,000 female patients. In both graphs, the blue curves represent nonresponders who had successfully converted to responders with the combination treatment, while the red curves represent nonresponders who did not demonstrate improvement with the addition of RIl12. Notice the increased presence of blue lines curving downward in the female population, indicating cancer cell regression. Also notice the lower average initial cancer cell populations in the female population than the male population.

These results appear to suggest that female patients may benefit slightly more from RIL12–nivolumab combination therapy than their male counterparts. These results are further supported by examining the temporal evolution of cancer cell counts in male and female patients during the combination treatment, which are depicted in Figure 6. In this figure, blue curves correspond to patients who converted to responders, while red curves correspond to those who did not. The plots indicate that, in the female population, those who converted into responders more often exhibited a larger decrease in cancer cell count 72 h after nivolumab administration, compared to males.

## 4. Discussion

Systems biology methods provide a safe and effective avenue for exploring the potential mechanisms that drive or inhibit a favorable patient response to cancer treatments, including targeted immunotherapies. While a mathematical framework may not manage to fully capture the intricacies of the human immune system, its representation of the major immune processes and interactions between several cell subtypes and signaling molecules allows for the discrete analysis of a treatment’s effects, and the identification of macroscopic parameter ranges that are most conducive to treatment response. With this knowledge, it may be possible to recommend a patient-specific therapy and to parameterize a model by examining an individual patient’s specific tumor-immune microenvironment, e.g., via tumor biopsy, to ultimately optimize individual patient treatment protocols.

With the help of experimental calibration and literature-based data, systems biology models can populate a significant patient cohort for the large-scale analysis of specific patient subgroups. This study focused on examining the effect of sex-specific immune differences on the response to nivolumab, an anti-PD-1 immune checkpoint inhibitor. Thus, large populations reflecting male and female HNSCC patients were generated in order to produce meaningful insight into the differences in treatment responses between the sexes and the origin of these differences. Such insight may prove useful for improving treatment outcomes for all patients, particularly for females, who tend to exhibit a lower response rate to cancer immunotherapy [13,14].

In this work, a thorough literature review was conducted to better understand the differences in the immune system between male and female patients and to recapitulate these differences in a systems biology model. Contextually relevant parameters were then subjected to a sensitivity analysis to identify a subset of nine parameters Ω that ultimately served to differentiate between male and female patients during the generation of virtual populations. Due to limited data in the literature that could be used to quantify the distinct ranges of individual kinetic parameters that are appropriate for male and female patients, we took the nominal ranges that were previously reported [12] to represent the sampling ranges for male virtual patients. We then updated the sampling ranges of the sex-specific parameters Ω as appropriate to generate representative female virtual patients that led to sex-specific differences in the immune cell populations that are consistent with those reported in the literature. In the simulations of nivolumab treatment, female virtual patient populations were consistently less responsive to treatment than the male population, with the response rates decreasing as the parameter sampling ranges were adjusted in the direction of “female” extremes. This suggests that, consistent with the literature, female patients exhibit a lower response to nivolumab than their male counterparts. Indeed, certain sex-specific characteristics described in Table 1 may reasonably lead to a decreased response rate when placed within the context of the network in Figure 1. For instance, higher PD-L1 and PD-1 expression in females will lead to an increased amount of PD-1:PD-L1 complex, which can further inhibit T-cell activity. Moreover, decreased CD8+ T-cell differentiation combined with a lower basal fraction of CD8+ naive T-cells negatively impacts cytotoxic functions that help kill cancer cells. Given the particularly high impact of the parameter λ (which is the per-cell expression of PD-L1) on the response rate, it is possible that the higher PD-L1 expression in females is the main contributor to the lower response rate that is observed clinically. However, further investigation is warranted to better understand the mechanisms driving female-specific nonresponse to immunotherapy, and whether this is directly related to other physiological differences, such as sex hormone levels [5].

Patients who do not respond to nivolumab monotherapy may benefit from combination therapy with recombinant IL12, which can be administered prior to nivolumab to boost the overall response to treatment. During this analysis, one large male population and one large female patient population were generated to examine the sex-specific differences in responses to the combination therapy, where the female population was generated by sampling from more extreme parameter ranges than for the nivolumab monotherapy. This enabled the consideration of a high degree of patient variability in the tumor-immune microenvironment. Subjecting both populations to several simulated treatment scenarios comprising a 10 pg/mL dose of RIL12 given at a specific time (ranging between 0 and 24 h) prior to nivolumab treatment revealed an apparent larger benefit for female nonresponders than male nonresponders, with a much higher percentage of females converted into responders due to RIL12. These results may provide a rationale for future research, perhaps in a clinical setting, into the combination of RIL12 with nivolumab in patients who do not respond to nivolumab alone. However, given the extremity of the female virtual patient population parameters and the limited literature information available, it remains to be tested whether female patients benefit more from RIL12 pre-treatment than male patients. Nevertheless, in the simulations, both sexes exhibited more favorable treatment outcomes when the RIL12 was dosed further in advance of the start of the nivolumab treatment schedule.

While some interesting results have emerged from this study, it is important to note that there are several limitations to this work. For example, the lack of quantitative data that were available in the literature to accurately parameterize sex-specific differences in the model parameters meant that we had to change the parameters by trial-and-error and look at their downstream effect on the immune landscape and response to treatment. This lack of data could have potentially adversely impacted the accuracy of our results, as it influenced our choice to include parameter variations that reflect sex-specific differences observed in different types of cancer. Moreover, given that the model may be non-identifiable, it is possible that several distinct parameter sets can lead to similar responses to treatment; and it is not straightforward to definitively state whether the sampling ranges used for female patients are in a biologically relevant range. As such, the results comparing responses to both the nivolumab treatment schedule and the combination therapy with recombinant IL12 may be misleading. This issue may be partially overcome by the collection and analysis of tumor biopsies for a larger cohort of male and female patients, under both control and treatment conditions. On that note, it is important to point out that the data that were collected to parameterize the sampling ranges for the male patient populations consisted of tumor biopsies from just 50 HNSCC patients. Therefore, the variability in the measured data may not be reflective of the total degree of variability in the tumor-immune microenvironment exhibited by patients. Finally, to remain tractable, several simplifications were made in the mathematical model, such as assumptions that PD-1 and PD-L1 are expressed uniformly across all T-cell populations and that all proteins achieve steady-state values instantly. Indeed, in reality, PD-1 and PD-L1 may be expressed differentially among distinct T-cell populations, and they may be regulated different by different sex hormones [5]. Therefore, the mathematical model does not capture the full complexities of the immune processes and immunomodulatory effects of treatment. Further expansion of the model to an in vivo setting, which includes drug distribution and pharmacokinetics, would be warranted before any clinical decisions are made based on the results of the simulations. Validation with clinical data may also serve as an important future step in this work.

As demonstrated by this study, systems biology approaches are generalizable to other contexts and can be used to provide valuable insights into the immunomodulatory effects of a variety of treatments, and for other patient populations. For example, future studies can be performed in a similar fashion to increase the understanding of nivolumab’s efficacy among patients of different age groups, perhaps taking into account additional underlying immune conditions that may affect the response to treatment. Moreover, the results of this work have paved a path to better understand the immune characteristics associated with female patients’ apparent resistance to treatment when compared to males, which may inform the advent of targeted combination treatments and/or alternative treatment schedules that can help increase treatment efficacy for females. Additionally, the results of the recombinant IL12 simulations provide a rationale for future clinical investigations into the impact of timing during combination treatment administration. While this analysis focused strictly on the efficacy of the treatment for eradicating cancer cells, future endeavors may include the analysis of other system characteristics that are related to treatment toxicity, such as cytokine release dynamics.

## Figures and Tables

**Table 1:** Sex-specific differences in basal immune system reported in the literature and associated model parameter(s) that were modified to capture the respective sex differences.

Sex Difference	Associated Parameter(s) Name (Description)	Reference
Increased IFNγ production by CD4+ T-cells in females	$p_{1-IFN}$ (rate of production of IFNγ by $Th_{1}$ cells)	[34]
Higher activated and proliferating CD4+ T-cells in females	$n_{4}$ (net proliferation rate of $T_{N4}$)	[33]
Higher activated and proliferating CD8+ T-cells in females	$n_{8}$ (net proliferation rate of $T_{N8}$), $n_{c}$ (IL12-independent net proliferation rate of $T_{c}$ cells)	[33]
Higher PD-1 expression on CD4+ T-cells in females	ρ (per-cell expression level of PD-1)	[35]
Higher PD-1 expression on CD8+ T-cells in males	ρ (per-cell expression level of PD-1)	[37]
High PD-L1 expression more likely on female tumors than male	λ (per-cell expression level of PD-L1), $\lambda_{Can-IFN}$ (IFNγ-dependent PD-L1 expression per cancer cell)	[36]
Increased $Th_{2}$ function in females	$p_{2-4}$ (rate of IL6-independent production of IL4 by Th2 cells), $p_{2-6}$ (rate of IL6 production by Th2 cells)	[33]
Increased $Th_{1}$ function in males	$n_{1}$ (net proliferation rate of $T_{H1}$ cells)	[33]
Higher IFNγ production by CD8+ in females	$p_{c_{I}FN}$ (rate of IFNγ production by $T_{c}$) cells	[37]
Increased CD8+ cytotoxic activity in females	$d_{c}$ (rate of differentiation of $T_{N8}$ cells into $T_{c}$ cells)	[33]
Higher CD4:CD8 ratio in females	Tc fraction (fraction of T-cells that are cytotoxic), TN8 fraction (fraction of T-cells that are CD8+ naive)	[38]

## Data Availability

The data presented and analyzed in this study are available from the corresponding author on reasonable request.

## Supplementary Materials

The following supporting information can be downloaded at: https://www.mdpi.com/article/10.3390/biom14121513/s1, Table S1: A description of the kinetic parameters in the mathematical model, Table S2: The values and ranges of the kinetic parameters, initial protein levels, and initial T-cell populations used for virtual patient simulation and local/global sensitivity analysis. References [11,12,17,18,19,20,21,22,23,24,25,26,27,28,29,30,31,32] are cited in the Supplementary Materials.
