# Supplementary material for: Sex-Related Differences in the Immune System Drive Differential Responses to Anti-PD-1 Immunotherapy
Source: Biomolecules. 2024 Nov 27;14(12):1513. doi: 10.3390/biom14121513 (PMC11673333; doi:10.3390/biom14121513)
Supplement: Supplementary file 1 [file biomolecules-14-01513-s001.zip › biomolecules-3243099-supplementary.pdf]

Supplementary information: Sex-related differences in the immune system drive differential responses to anti-PD-1 immunotherapy

Sonja Cotra[1], Mohammad Kohandel[1], Michelle Przedborski[1]

[1]Department of Applied Mathematics, University of Waterloo
November 13, 2024

This supplementary file discusses a systems biology model developed from prior work [16, 11] to gauge sex differences in regards to the administration of nivolumab, an immune checkpoint inhibitor, as well as recombinant IL12 combination therapy. Before going into the details regarding the methodology and results of this project, we will first provide in-depth information regarding the equations and parameters that inform this model.

1 Equations

1.1 Modeling nivolumab administration

In order to capture the dynamics of nivolumab treatment within a patient's immune system, several equations were created with the aim of capturing tumoral response in an ex vivo experimental setup. With a system of 15 coupled ordinary differential equations mathematically describing nivolumab administration and another 5 pertaining specifically to recombinant IL12 dynamics, a system is built comprising of immunotherapy drugs, cancer cells, and immune cells. Immune cell proliferation in these equations is assumed to represent mitosis, with both proliferation and death rates proporitonal to the population of the respective cellular species.

1. Time evolution of naive helper (CD4+) T-cell population:

$$\begin{aligned}\frac{dT_{N4}}{dt} &= n_4 T_{N4} - \left(d_{1-12} T_{N4} \frac{[IL-12]}{q_{dIL12} + [IL-12]}\right.\\ &\quad \left. + d_{1-IFN} T_{N4} \frac{[IFN\gamma]}{q_{IFN-1} + [IFN\gamma]}\right)\left(\frac{s_1}{s_1 + [PD-1:PD-L1]}\right) \\ &\quad - \left(d_2 T_{N4} \frac{[IL-4]}{q_{dIL4} + [IL-4]}\right)\left(\frac{s_2}{s_2 + [PD-1:PD-L1]}\right)\end{aligned} \tag{1}$$

Net proliferation of T_{N4} cells is illustrated by the first term. The second term describes the differentiation of T_{N4} cells into Th_1 cells in the presence of IL-12 [19], while the third term described the same process in the presence of IFNγ [2]. These differentiation processes are

inhibited by the PD-1:PD-L1 complex [5, 10, 17], as indicated by the multiplicative factor applied to terms 2 and 3. The fourth and final term describes the differentiation of T_{N4} cells into Th_2 cells in the presence of IL-4 [2, 19], which is also inhibited by the PD-1:PD-L1 complex [5, 10, 17].

2. Time evolution of type 1 helper T-cell population:

$$\frac{dTh_1}{dt} = n_1 Th_1 + \left(d_{1-12} T_{N4} \frac{[IL-12]}{q_{dIL12} + [IL-12]} + d_{1-IFN} T_{N4} \frac{[IFN\gamma]}{q_{IFN-1} + [IFN\gamma]} \right) \left(\frac{s_1}{s_1 + [PD-1:PD-L1]} \right) \tag{2}$$

The first term describes the net proliferation of Th_1 cells. The remaining terms, similarly to terms 2 and 3 in Equation (1) describe the increase in the Th_1 cell population due to the differentiation of T_{N4} cells into Th_1 cells in the presence of IL-12 [19] (term 2) and IFNγ [2] (term 3), which is inhibited by the PD-1:PD-L1 complex [5, 10, 17].

3. Time evolution of type 2 helper T-cell population:

$$\frac{dTh_2}{dt} = \left(g_2 Th_2 + g_{2-4} Th_2 \frac{[IL-4]}{q_{gIL4} + [IL-4]} \right) \left(\frac{r_{IFN}}{r_{IFN} + [IFN\gamma]} \right) + \left(d_2 T_{N4} \frac{[IL-4]}{q_{dIL4} + [IL-4]} \right) \left(\frac{s_2}{s_2 + [PD-1:PD-L1]} \right) - \delta_2 Th_2 \tag{3}$$

The first term describes the proliferation of Th_2 cells, with the second term denoting upregulation by IL-4 [19, 2]. This proliferation is also inhibited by IFNγ [3] as seen by the inhibition factor that is applied to the first two terms. The third term describes the increase in Th_2 population resulting from the differentiation of T_{N4} cells into Th_2 cells in the presence of IL-4 [2, 19], which is inhibited by the PD-1:PD-L1 [5, 10, 17] complex. The final term describes naturally occurring death of the Th_2 cells.

4. Time evolution of naive cytotoxic (CD8+) T-cell population:

$$\frac{dT_{N8}}{dt} = n_8 T_{N8} - d_c T_{N8} \left(\frac{Th_1}{q_1 + Th_1} \right) \left(\frac{s_C}{s_C + [PD-1:PD-L1]} \right) \tag{4}$$

The first term describes the net proliferation of T_{N8} cells. The second term describes the process of T_{N8} cells differentiating into T_c cells, as motivated by the company of Th_1 cells[15, 12]. This is also inhibited by the PD-1:PD-L1 complex [5, 10, 17].

5. Time evolution of cytotoxic (CD8+) T-cell population:

$$\frac{dT_c}{dt} = n_c T_c + g_{c-12} T_c \frac{[IL-12]}{q_{gIL12} + [IL-12]} + d_c T_{N8} \left(\frac{Th_1}{q_1 + Th_1} \right) \left(\frac{s_c}{s_c + [PD-1:PD-L1]} \right) \tag{5}$$

The first term describes the net proliferation of T_c cells. This process is upregulated by IL-12 [7], as demonstrated by the second term. The third term describes the increase in the T_c cell population that occurs when the T_{N8} cells differentiate into T_c cells, which in turn happens in the presence of Th_1-activated dendritic cells (DCs)[15, 12]. Finally, this differentiation process is inhibited by the PD-1:PD-L1 complex [5, 10, 17].

6. Time evolution of cancer cell population:

$$\frac{dC}{dt} = n_{Can}C - k_c C T_c \tag{6}$$

The first term describes the net proliferation of cancer cells. The second term describes the killing of cancer cells by T_c cells, which can occur via mechanisms such as granzyme/perforin-induced apoptosis [5, 18].

7. Time evolution of IFNγ concentration:

$$\frac{d[IFN\gamma]}{dt} = p_{1-IFN}Th_1\left(\frac{r_{IL4}}{r_{IL4} + [IL-4]}\right)\left(\frac{r_{IL6}}{r_{IL6} + [IL-6]}\right) + p_{c-IFN}T_c - \delta_{IFN}[IFN\gamma] \tag{7}$$

The first term describes the secretion of IFNγ by Th_1 cells [3, 19, 2], which is inhibited by IL-4 [4] and IL-6 [2]. The second term describes the secretion of IFNγ by T_c cells [5], and the third term describes the natural decay of IFNγ.

8. Time evolution of IL-4 concentration:

$$\frac{d[IL-4]}{dt} = p_{2-4}Th_2 + p_{2-4-6}Th_2\left(\frac{[IL-6]}{q_{IL6} + [IL-6]}\right) - \delta_{IL4}[IL-4] \tag{8}$$

The first term describes the IL-4 secreted by Th_2 cells [3, 19, 2]. The second term describes the additional secretion of IL-4 by Th_2 cells in the company of IL-6 [14, 2]. The third term describes the natural decay of IL-4.

9. Time evolution of IL-6 concentration:

$$\frac{d[IL-6]}{dt} = p_{2-6}Th_2 + p_{Can-6}C - \delta_{IL6}[IL-6] \tag{9}$$

The first term describes the secretion of IL-6 by Th_2 cells [3]. The second term describes the production of IL-6 by ntigen presenting cells,[13, 2] which is assumed to be directly proportional to the number of cancer cells (term 2). The third term describes the natural decay of IL-6.

10. Time evolution of IL-12 concentration:

$$\frac{d[IL-12]}{dt} = p_{Can-12}C + p_{1-12}Th_1 - \delta_{IL12}[IL-12] \tag{10}$$

The first term describes the production of IL-12 by DCs, which is also assumed to be directly proportional to the number of cancer cells [13]. The second term describes the further production of IL-12 by Th_1-activated DCs [8]. The third term describes the natural decay of IL-12.

11. PD-1 concentration and its time evolution:

$$[PD-1] = \rho\left(Th_1 + Th_2 + T_c\right) \tag{11}$$

$$\frac{d[PD-1]}{dt} = \rho\left(\frac{dTh_1}{dt} + \frac{dTh_2}{dt} + \frac{dT_c}{dt}\right) - \beta_+[PD-1][PD-L1] + \beta_-[PD-1:PD-L1] - \alpha_+[PD-1][A] + \alpha_-[A:PD-1] \tag{12}$$

All activated T-cells, i.e. Th_1, Th_2, and T_c, express PD-1 [5, 17], so the total concentration of PD-1 is proportional to the sum of the T-cell populations, as indicated by Equation (11). For simplification purposes, we assume that the same amount of PD-1 is expressed evenly on all types of T-cells, thus allowing for equal proportionality constants for each T-cell population.

Equation (12) describes the time evolution of PD-1. The first three terms describe the change in the PD-1 levels due to changing Th_1, Th_2, and T_c populations, respectively. The fourth term describes the binding of PD-1 to PD-L1 to form the PD-1:PD-L1 complex, while the fifth term describes the dissociation of this complex [5]. Similarly,the fifth term described the binding of PD-1 to Nivolumab and the sixth term describes its dissociation [17].

12. PD-L1 concentration and its time evolution:

$$[PD-L1] = \lambda\left(Th_1 + Th_2 + T_c + C\right) + \lambda_{Can-IFN} C\left(\frac{[IFN\gamma]}{q_{IFN-PDL1} + [IFN\gamma]}\right) \tag{13}$$

$$\begin{aligned}\frac{d[PD-L1]}{dt} &= \lambda\left(\frac{dTh_1}{dt} + \frac{dTh_2}{dt} + \frac{dT_c}{dt} + \frac{dC}{dt}\right) \\ &+ \lambda_{Can-IFN}\frac{dC}{dt}\left(\frac{[IFN\gamma]}{q_{IFN-PDL1} + [IFN\gamma]}\right) \\ &- \beta_+[PD-1][PD-L1] + \beta_-[PD-1:PD-L1]\end{aligned} \tag{14}$$

Like PD-1, PD-L1 is also expressed on all activated T cells, i.e. Th_1, Th_2, T_c [5, 10]. It is also expressed on cancer cells [5, 17, 10], leaving the total concentration of PD-L1 proportional, in part, to the sum of the T-cell and cancer cell populations, as indicated by the first four terms in Equation (13). We make the same simplifying assumption as before that the PD-L1 expression is evenly distributed across all types of cells. Furthermore, the expression of PD-L1 by cancer cells specifically experiences upregulation by IFNγ [5, 17, 10], which is illustrated by a factor on the fifth term in Equation (13).

The time evolution of PD-L1 is described by Equation (14). As such, the first five terms describe the change in PD-L1 levels that occur due to changing T-cell and cancer cell populations, owing to the aforementioned proportionality. We simplify the model by assuming that the proteins reach their steady state values instantaneously with respect to the time scale of the changes in cell populations (i.e. the cell division rate) so that $\frac{d[IFN\gamma]}{dt} \approx 0$. The seventh term describes the binding of PD-1 to PD-L1, while the dissociation of the resulting PD-1:PD-L1 complex [5] is described by the seventh term.

13. Time evolution of PD-1:PD-L1 complex concentration:

$$\frac{d[PD-1:PD-L1]}{dt} = \beta_+[PD-1][PD-L1] - \beta_-[PD-1:PD-L1] \tag{15}$$

The first term describes the formation of the PD-1:PD-L1 complex via the binding of PD-1 to PD-L1, and the second term describes the dissociation of this complex [5].

14. Time evolution of free Nivolumab concentration:

$$\frac{d[A]}{dt} = \tilde{A}(t) - \alpha_+[A][PD-1] + \alpha_-[A:PD-1] - \delta_A[A] \tag{16}$$

The first term describes the administration of Nivolumab into the system, which can be time-dependent in certain treatment schedules. The second term describes the binding of PD-1 to Nivolumab, thus forming the Nivolumab:PD-1 complex, and the third term describes the dissociation of this complex [17]. We assume that the dissociation constant $K_\alpha \equiv \alpha_-/\alpha_+ \ll K_\beta \equiv \beta_-/\beta_+$ so that Nivolumab has a higher binding affinity for PD-1 than that of PD-L1, allowing the drug to displace PD-L1 from the PD-1:PD-L1 complex, dismantling it. In simulations, we assume that the rate of association of PD-1 is equivalent for Nivolumab and PD-L1, i.e. $\alpha_+ = \beta_+$, which ends up removing a kinetic parameter from the system, as will be seen in the next section. The fourth term in the equation describes the natural decay of Nivolumab.

15. Time evolution of Nivolumab:PD-1 complex concentration:

$$\frac{d[A:PD-1]}{dt} = \alpha_+[A][PD-1] - \alpha_-[A:PD-1] \tag{17}$$

The first term describes the binding of Nivolumab with PD-1 to form the Nivolumab:PD-1 complex, and the second term describes its dissociation [17].

1.2 Modeling recombinant IL12 administration

Logically, the administration of recombinant IL-12 should ideally increase the total IL-12 concentration in the system, directly affecting the time evolution of the populations of naive helper (CD4+) T-cells, type 1 helper T-cells, and CD8+ cytotoxic T-cells. These effects are described by the following five equations. All other equations remain identical to those presented above in the preceding section.

1. We assume that the initial recombinant IL-12 dose experiences natural decay. Hence, the time evolution of recombinant IL-12, $[R-IL12]$, may be described by:

$$\frac{d[R-IL12]}{dt} = -\delta_{R-IL12}[R-IL12], \tag{18}$$

where the decay rate is taken to correspond to a drug half-life of 30 hours [1].

2. We then define the total IL-12 concentration, $[T-IL12]$, as:

$$[T-IL12] = [R-IL12] + [IL-12], \tag{19}$$

where the time evolution of $[IL-12]$ is given in Equation 10 of Section 1.

Then all instances of IL-12 concentration in the equations for the naive helper (CD4+) T-cells, type 1 helper T-cells, and CD8+ cytotoxic T-cells are replaced by the total IL-12 concentration, resulting in the following equations:

3. Time evolution of naive helper (CD4+) T-cell population:

$$\frac{dT_{N4}}{dt} = n_4 T_{N4} - \left(d_{1-12} T_{N4} \frac{[T-IL12]}{q_{dIL12} + [T-IL12]} \right. \tag{20}$$

$$\left. + d_{1-IFN} T_{N4} \frac{[IFN\gamma]}{q_{IFN-1} + [IFN\gamma]} \right) \left(\frac{s_1}{s_1 + [PD-1:PD-L1]} \right)$$

$$- \left(d_2 T_{N4} \frac{[IL-4]}{q_{dIL4} + [IL-4]} \right) \left(\frac{s_2}{s_2 + [PD-1:PD-L1]} \right) \tag{21}$$

4. Time evolution of type 1 helper T-cell population:

$$\frac{dTh_1}{dt} = n_1 Th_1 + \left(d_{1-12} T_{N4} \frac{[T-IL12]}{q_{dIL12}} \right. \tag{22}$$

$$\left. + [T-IL12] + d_{1-IFN} T_{N4} \frac{[IFN\gamma]}{q_{IFN-1} + [IFN\gamma]} \right) \left(\frac{s_1}{s_1 + [PD-1:PD-L1]} \right) \tag{23}$$

5. Time evolution of cytotoxic (CD8+) T-cell population:

$$\frac{dT_c}{dt} = n_c T_c + g_{c-12} T_c \frac{[T-IL12]}{q_{gIL12} + [T-IL12]} + d_c T_{N8} \left(\frac{Th_1}{q_1 + Th_1} \right) \left(\frac{s_c}{s_c + [PD-1:PD-L1]} \right). \tag{24}$$

2 Parameters

As shown in the previously displayed equations, this mathematical model relies on several parameters. We provide a description for each of the 47 relevant kinetic parameters in the following table.

Table S1: Description of the kinetic parameters in the mathematical model

**Name**	**Description**
n_4	Net proliferation rate of T_{N4} cells
n_8	Net proliferation rate of T_{N8} cells
n_1	Net proliferation rate of Th_1 cells
n_c	IL12-independent net proliferation rate of T_c cells
n_{Can}	Net proliferation rate of cancer cells
g_2	IL4-independent growth rate of Th_2 cells
g_{2-4}	IL4-dependent growth rate of Th_2 cells
g_{c-12}	IL12-dependent growth rate of T_c cells

δ_2	Death rate of Th_2 cells
d_{1-IFN}	IFNγ-dependent differentiation rate of T_{N4} cells into Th_1 cells
d_{1-12}	IL12-dependent differentiation rate of T_{N4} cells into Th_1 cells
d_2	IL4-dependent differentiation rate of T_{N4} cells into Th_2 cells
d_c	Rate of differentiation of T_{N8} cells into T_c cells
k_c	Rate of cancer cell killing by T_c cells
p_{1-IFN}	Rate of production of IFNγ by Th_1 cells
p_{2-4-6}	IL6-dependent production of IL-4 by Th_2 cells
p_{Can-6}	Rate of production of IL-6 by antigen presenting cells (assumed proportional to the number of cancer cells)
p_{Can-12}	Rate of production of IL-12 by DCs (assumed proportional to the number of cancer cells)
δ_{IFN}	Decay rate of IFNγ
δ_{IL4}	Decay rate of IL-4
δ_{IL6}	Decay rate of IL-6
δ_{IL12}	Decay rate of IL-12
δ_A	Decay rate of Nivolumab
q_1	Half-maximal Th_1 cell population for T_{N8} differentiation into T_c cells
q_{IFN-1}	Half-maximal IFNγ concentration for IFNγ-dependent differentiation of T_{N4} cells into Th_1 cells
$q_{IFN-PDL1}$	Half-maximal IFNγ concentration for IFNγ-dependent PD-L1 expression by cancer cells
q_{gIL4}	Half-maximal IL-4 concentration for IL4-dependent proliferation of Th_2 cell
q_{dIL4}	Half-maximal IL-4 concentration for IL4-dependent differentiation of T_{N4} cells into Th_2 cells
q_{IL6}	Half-maximal IL-6 concentration for IL6-dependent production of IL-4 by Th_2 cells
q_{dIL12}	Half-maximal IL-12 concentration for IL12-dependent differentiation of T_{N4} cells into Th_1 cells
q_{gIL12}	Half-maximal IL-12 concentration for IL12-dependent proliferation of T_c cells
r_{IFN}	Half-maximal IFNγ concentration for IFNγ-dependent inhibition of Th_2 proliferation
r_{IL4}	Half-maximal IL-4 concentration for IL4-dependent inhibition of IFNγ production by Th_1 cell
r_{IL6}	Half-maximal IL-6 concentration for IL6-dependent inhibition of IFNγ production by Th_1 cells
ρ	Per-cell expression level of PD-1

λ	Per-cell expression level of PD-L1
$\lambda_{Can-IFN}$	IFNγ-dependent PD-L1 expression per cancer cell
β_+	Rate of association of PD-1 and PD-L1
β_-	Rate of dissociation of PD-1:PD-L1 complex
α_-	Rate of dissociation of Nivolumab:PD-1 complex
s_1	Half-maximal PD-1:PD-L1 concentration for inhibition of T_{N4} differentiation into Th_1 cells
s_2	Half-maximal PD-1:PD-L1 concentration for inhibition of T_{N4} differentiation into Th_2 cell
s_c	Half-maximal PD-1:PD-L1 concentration for inhibition of T_{N8} differentiation into T_c cells
p_{1-12}	Rate of IL-12 production by Th_1 cells
p_{2-4}	Rate of IL6-independent production of IL-4 by Th_2 cells
p_{2-6}	Rate of IL-6 production by Th_2 cells
p_{c-IFN}	Rate of IFNγ production by T_c cells

We display the numerical values of the parameters and initial conditions for protein levels and relative T-cell populations in the following Table S2. Average patient data is represented by the "Nominal value" column and the parameter units can be seen in the "Units" column. Note that the unit "min" denotes a timescale of minutes in an abbreviated form. We also present ranges in the "Range" column for each parameter with the purpose of searching the parameter space with Matlab's genetic algorithm to match the average patient data. For protein levels and T-cell fractions, the ranges were set by the minimum and maximum experimentally measured values from all patient data. All of these ranges were used during global sensitivity analysis. Additionally, these ranges were used to simulate virtual patient data for male populations subjected to a 3-day nivolumab treatment schedule. Note that female populations were generated with some of these ranges altered by applying random factors to each end of the range. When using the genetic algorithm to match the average patient data, the T-cell fractions were set to the average of all patients without treatment while protein levels were sampled from a range set within one standard deviation of the average value.

Table S2: Values and ranges of the kinetic parameters, initial protein levels, and initial T-cell popu-lations used for virtual patient simulation and local/global sensitivity analysis

**Parameter**	**Nominal value**	**Range**	**Units**	**Reference**
n_4	2.9×10^{-2}	$\ln(2)/20 - \ln(2)$	day^{-1}	estimated from [3, 4]
n_8	8.2×10^{-3}	$\ln(2)/20 - \ln(2)$	day^{-1}	estimated from [3, 4]

n_1	7.7×10^{-3}	$\ln(2)/20 - \ln(2)$	day^{-1}	estimated from [3, 4]
n_c	8.3×10^{-3}	$\ln(2)/20 - \ln(2)$	day^{-1}	estimated from [3, 4]
n_{Can}	6.9×10^{-2}	$\ln(2)/100 - \ln(2)/5$	day^{-1}	estimated
g_2	3.7×10^{-2}	$\ln(2)/20 - \ln(2)$	day^{-1}	estimated from [3, 4]
g_{2-4}	3.9×10^{-2}	$\ln(2)/20 - \ln(2)$	day^{-1}	estimated from [3, 4]
g_{c-12}	3.8×10^{-2}	$\ln(2)/20 - \ln(2)$	day^{-1}	estimated from [3, 4]
δ_2	1.2×10^{-2}	$\ln(2)/60 - \ln(2)/7$	day^{-1}	estimated from [3, 4]
d_{1-IFN}	7.4×10^{-2}	$\ln(2)/20 - \ln(2)$	day^{-1}	estimated from [9]
d_{1-12}	7.4×10^{-2}	$\ln(2)/20 - \ln(2)$	day^{-1}	estimated from [9]
d_2	3.6×10^{-2}	$\ln(2)/20 - \ln(2)$	day^{-1}	estimated from [9]
d_c	3.7×10^{-2}	$\ln(2)/20 - \ln(2)$	day^{-1}	estimated from [9]
k_c	3.6×10^{-4}	$10^{-8} - 10^{-2}$	T_c cell$^{-1}\cdot$ day^{-1}	estimated
p_{1-IFN}	8.9×10^{-4}	$6.5 \times 10^{-4} - 1.7 \times 10^{-2}$	$\frac{\text{pg/mL}}{Th_1 \text{ cell} \cdot \text{day}}$	estimated
p_{2-4-6}	3.9×10^{-4}	$1.4 \times 10^{-7} - 1.4 \times 10^{-2}$	$\frac{\text{pg/mL}}{Th_2 \text{ cell} \cdot \text{day}}$	estimated
p_{Can-6}	8.9×10^{-3}	$7.2 \times 10^{-3} - 7.2 \times 10^{-1}$	$\frac{\text{pg/mL}}{\text{cancer cell} \cdot \text{day}}$	estimated
p_{Can-12}	1.3×10^{-6}	$8.3 \times 10^{-7} - 9.7 \times 10^{-6}$	$\frac{\text{pg/mL}}{\text{cancer cell} \cdot \text{day}}$	estimated from [6]
δ_{IFN}	7.0×10^{-4}	$\ln(2)/1000 - \ln(2)/60$	min^{-1}	estimated from [4]
δ_{IL4}	7.7×10^{-4}	$\ln(2)/1000 - \ln(2)/60$	min^{-1}	estimated from [4]
δ_{IL6}	7.0×10^{-4}	$\ln(2)/1000 - \ln(2)/60$	min^{-1}	estimated from [4]
δ_{IL12}	4.8×10^{-4}	$\ln(2)/1440 - \ln(2)/600$	min^{-1}	estimated from [6]

δ_A	4.7×10^{-2}	$\ln(2)/15 - \ln(2)/10$	day^{-1}	estimated from [6]
q_1	1.7×10^{2}	$1 - 10^{5}$	Th_1 cells	estimated
q_{IFN-1}	8.6×10^{-1}	$10^{-3} - 10^{2}$	$[IFN\gamma]$ (pg/mL)	estimated
$q_{IFN-PDL1}$	4.1×10^{-1}	$10^{-3} - 10^{2}$	$[IFN\gamma]$ (pg/mL)	estimated
q_{gIL4}	1.8	$10^{-3} - 10^{3}$	$[IL-4]$ (pg/mL)	estimated
q_{dIL4}	2.4×10^{-2}	$10^{-3} - 10^{3}$	$[IL-4]$ (pg/mL)	estimated
q_{IL6}	1.5×10^{2}	$10^{2} - 10^{4}$	$[IL-6]$ (pg/mL)	estimated
q_{dIL12}	5.0×10^{-2}	$10^{-3} - 10^{2}$	$[IL-12]$ (pg/mL)	estimated
q_{gIL12}	2.0	$10^{-3} - 10^{2}$	$[IL-12]$ (pg/mL)	estimated
r_{IFN}	2.0×10^{-1}	$10^{-3} - 10^{2}$	$[IFN\gamma]$ (pg/mL)	estimated
r_{IL4}	3.3	$10^{-1} - 10^{3}$	$[IL-4]$ (pg/mL)	estimated
r_{IL6}	1.6×10^{2}	$10^{2} - 10^{4}$	$[IL-6]$ (pg/mL)	estimated
ρ	7.7×10^{-2}	$10^{-6} - 10^{1}$	(pg/mL)/T-cell	estimated from [6]
λ	1.0×10^{1}	$10^{-6} - 10^{1}$	(pg/mL)/cell	estimated from [6]
$\lambda_{Can-IFN}$	9.1×10^{-4}	$10^{-10} - 10^{-1}$	(pg/mL)/cancer cell	estimated from [6]
β_+	9.8×10^{-4}	$1.4 \times 10^{-4} - 1.4 \times 10^{-1}$	$\left((\text{pg/mL}) \cdot \text{day}\right)^{-1}$	estimated
β_-	2.5	$1.4 - 1.4 \times 10^{2}$	day^{-1}	estimated
α_-	1.5×10^{-3}	$1.4 \times 10^{-3} - 1.4 \times 10^{-1}$	$\left((\text{pg/mL}) \cdot \text{day}\right)^{-1}$	estimated
s_1	4.5×10^{-3}	$10^{-3} - 10^{5}$	$[PD-1:PD-L1]$ (pg/mL)	estimated
s_2	9.6×10^{-1}	$10^{-3} - 10^{5}$	$[PD-1:PD-L1]$ (pg/mL)	estimated
s_c	2.2	$10^{-3} - 10^{5}$	$[PD-1:PD-L1]$ (pg/mL)	estimated
p_{1-12}	2.4×10^{-2}	see text	$\frac{\text{pg/mL}}{Th_2 \text{ cell} \cdot \text{day}}$	–
p_{2-4}	3.1×10^{-3}	see text	$\frac{\text{pg/mL}}{Th_2 \text{ cell} \cdot \text{day}}$	–
p_{2-6}	3.9×10^{1}	see text	$\frac{\text{pg/mL}}{T_c \text{ cell} \cdot \text{day}}$	–
p_{c-IFN}	3.8×10^{-5}	see text	$\frac{\text{pg/mL}}{T_c \text{ cell} \cdot \text{day}}$	–
**Protein**	**Nominal value**	**Range**	**Units**	**Reference**

IFNγ	0.38	0.18 – 482.31	pg/mL	patient data
IL-12	1.76	1.82 – 11.44	pg/mL	patient data
IL-6	7626.67	149.15 – 35884.0	pg/mL	patient data
IL-4	0.62	0.10 – 61.37	pg/mL	patient data
**Cell fraction**	**Nominal value**	**Range**	**Units**	**Reference**
Cancer fraction	0.30	0.1 – 0.9*	–	estimated
TN8 fraction	0.65	0.21–0.97	–	patient data
Tc fraction	0.10	0.0–0.59	–	patient data
CD4+ fraction	0.25	0.01–0.69**	–	patient data
Th1 fraction	2.0×10^{-3}	0 – 0.99	–	estimated
Th2 fraction	7.9×10^{-3}	0 – 0.99***	–	estimated

*We assume that the tumor consists of a population of cancer cells and a population of immune cells. Then, corresponding to the nominal values given in Table 3, 81% of the tumor is cancer cells and the remaining 19% is the total immune cell population.
**The total immune cell population consists of naive CD8+ T-cells (T_{N8}), CD8+ cytotoxic T-cells (T_c), and a population of CD4+ cells, so we always impose the constraint (TN8 fraction + Tc fraction + CD4+ fraction) = 1.
***The CD4+ fraction is further subdivided, being comprised of naive helper CD4+ T-cells (T_{N4}), type 1 helper T-cells (Th_1) and type 2 helper T-cells (Th_2. As a result, impose the constraint (Th1 fraction + Th2 fraction + TN4 fraction) = 1 at all times.

Parameters 44-47 do not have a specified range as they were calculated at the beginning of each simulation under the assumption of initial steady state protein levels (Equations (7)-(10)) using the initial T-cell population values, along with the constraint that all parameters are non-negative. Thus for local and global sensitivity analysis, it was necessary to re-calculate parameters 44-47 for each simulation.

In order to ensure Nivolumab's increased binding affinity for PD-1 compared to that of PD-L1, we imposed the additional constraint stating that parameter 40 < 0.1 parameter 39 for each simulation.

Furthermore, we note that the PD-1 and PD-L1 concentrations were initialized for each simulation according to Equations (11) and (13), respectively, with the initial cell populations and relevant protein level. In an initial analysis, we used an increased upper bound for the net proliferation rate of cancer cells, parameter 5. This occasionally resulted in nonphysical growth of the cancer population over the three day treatment window in cases of non-response. When this happened, the model output was most sensitive to parameters controlling the CD8+ cytotoxic T-cell population and its efficiency at killing the cancer cells. Important notes regarding fraction parameters are situated below the table.

References

[1] V. Carreño, S. Zeuzem, U. Hopf, P. Marcellin, W. G. E. Cooksley, J. Fevery, M. Diago, R. Reddy, M. Peters, K. Rittweger, et al. A phase i/ii study of recombinant human interleukin-12 in patients with chronic hepatitis b. *Journal of hepatology*, 32(2):317–324, 2000.

[2] S. Diehl and M. Rincón. The two faces of il-6 on th1/th2 differentiation. *Molecular immunology*, 39(9):531–536, 2002.

[3] M. A. Fishman and A. S. Perelson. Th1/th2 cross regulation. *Journal of theoretical biology*, 170(1):25–56, 1994.

[4] M. A. Fishman and A. S. Perelson. Th1/th2 differentiation and cross-regulation. *Bulletin of mathematical biology*, 61(3):403–436, 1999.

[5] G. J. Freeman, E. J. Wherry, R. Ahmed, and A. H. Sharpe. Reinvigorating exhausted hiv-specific t cells via pd-1–pd-1 ligand blockade. *Journal of Experimental Medicine*, 203(10):2223–2227, 2006.

[6] X. Lai and A. Friedman. Combination therapy of cancer with cancer vaccine and immune checkpoint inhibitors: A mathematical model. *PLoS One*, 12(5):e0178479, 2017.

[7] W. Lasek, R. Zagożdżon, and M. Jakobisiak. Interleukin 12: still a promising candidate for tumor immunotherapy? *Cancer Immunology, Immunotherapy*, 63(5):419–435, 2014.

[8] S. E. Macatonia, N. A. Hosken, M. Litton, P. Vieira, C.-S. Hsieh, J. A. Culpepper, M. Wysocka, G. Trinchieri, K. M. Murphy, and A. O'Garra. Dendritic cells produce il-12 and direct the development of th1 cells from naive cd4+ t cells. *The Journal of Immunology*, 154(10):5071–5079, 1995.

[9] B. F. Morel, J. Kalagnanam, and P. A. Morel. Mathematical modeling of th1-th2 dynamics. In *Theoretical and experimental insights into immunology*, pages 171–190. Springer, 1992.

[10] T. Okazaki and T. Honjo. The pd-1–pd-l pathway in immunological tolerance. *Trends in immunology*, 27(4):195–201, 2006.

[11] M. Przedborski, M. Smalley, S. Thiyagarajan, A. Goldman, and M. Kohandel. Systems biology informed neural networks (sbinn) predict response and novel combinations for pd-1 checkpoint blockade. *Communications Biology*, 4(877), 2021.

[12] J. P. Ridge, F. Di Rosa, and P. Matzinger. A conditioned dendritic cell can be a temporal bridge between a cd4+ t-helper and a t-killer cell. *Nature*, 393(6684):474, 1998.

[13] M. Rincón, J. Anguita, T. Nakamura, E. Fikrig, and R. A. Flavell. Interleukin (il)-6 directs the differentiation of il-4–producing cd4+ t cells. *Journal of Experimental Medicine*, 185(3):461–470, 1997.

[14] S. Romagnani. The th1/th2 paradigm. *Immunology today*, 18(6):263–266, 1997.

[15] S. Sakaguchi. Regulatory t cells: key controllers of immunologic self-tolerance. *Cell*, 101(5):455–458, 2000.

[16] M. Smalley, M. Przedborski, S. Thiyagarajan, M. Pellowe, A. Verma, N. Brijwani, D. Datta, M. Jain, B. U. Shanthappa, V. Kapoor, et al. Integrating systems biology and an ex vivo human tumor model elucidates pd-1 blockade response dynamics. *Iscience*, 23(6), 2020.

[17] M. Sznol and L. Chen. Antagonist antibodies to pd-1 and b7-h1 (pd-l1) in the treatment of advanced human cancer—response. *Clinical Cancer Research*, 19(19):5542–5542, 2013.

[18] J. A. Trapani and M. J. Smyth. Functional significance of the perforin/granzyme cell death pathway. *Nature Reviews Immunology*, 2(10):735, 2002.

[19] A. Yates, C. Bergmann, J. L. Van Hemmen, J. Stark, and R. Callard. Cytokine-modulated regulation of helper t cell populations. *Journal of theoretical biology*, 206(4):539–560, 2000.
